# Supplementary figures and images for: Bioinformatics analysis for the role of CALR in human cancers
Source: PLoS One. 2021 Dec 15;16(12):e0261254. doi: 10.1371/journal.pone.0261254 (PMC8673678; doi:10.1371/journal.pone.0261254)

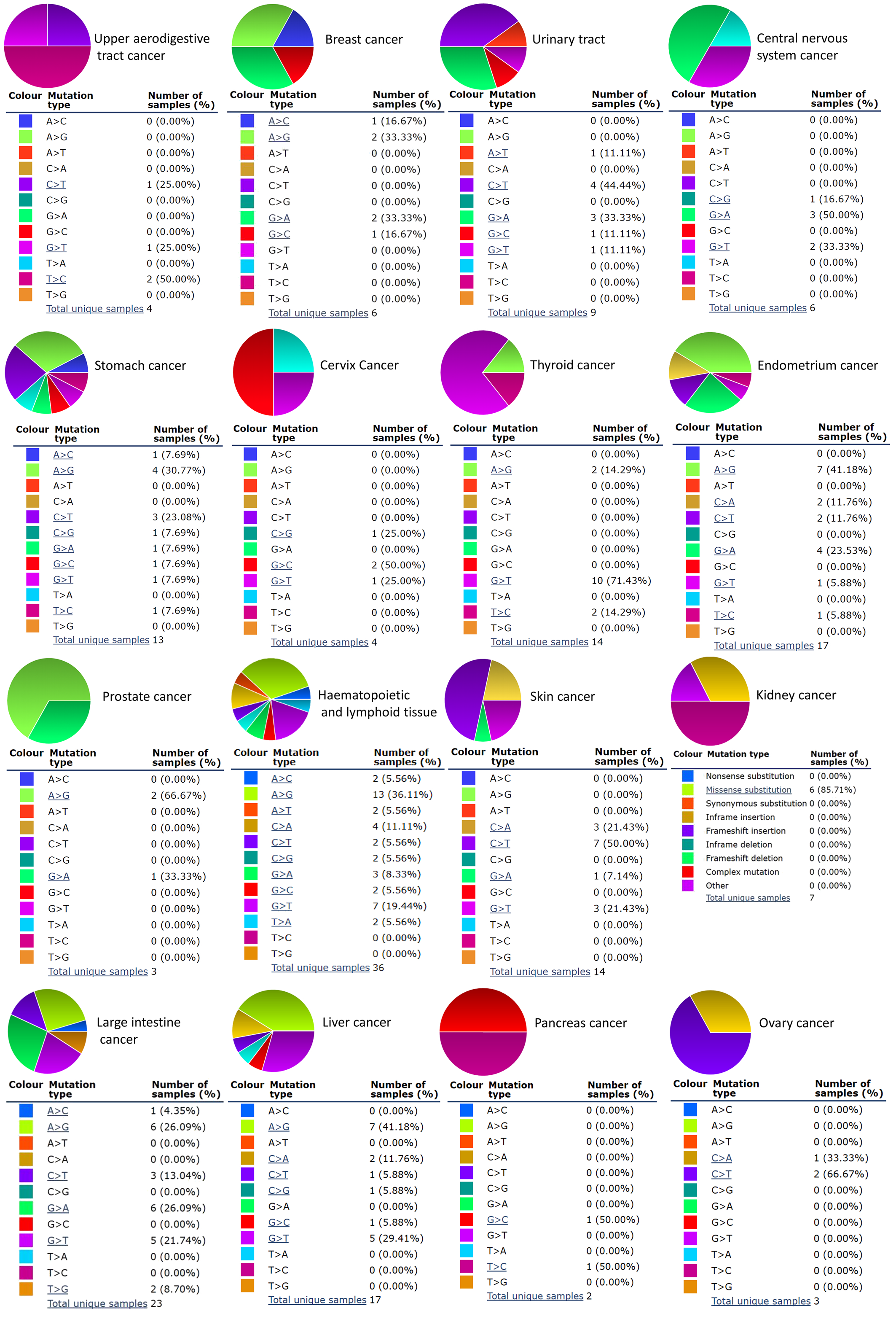

Supplement: S1 Fig — (TIF) [file pone.0261254.s001.tif]

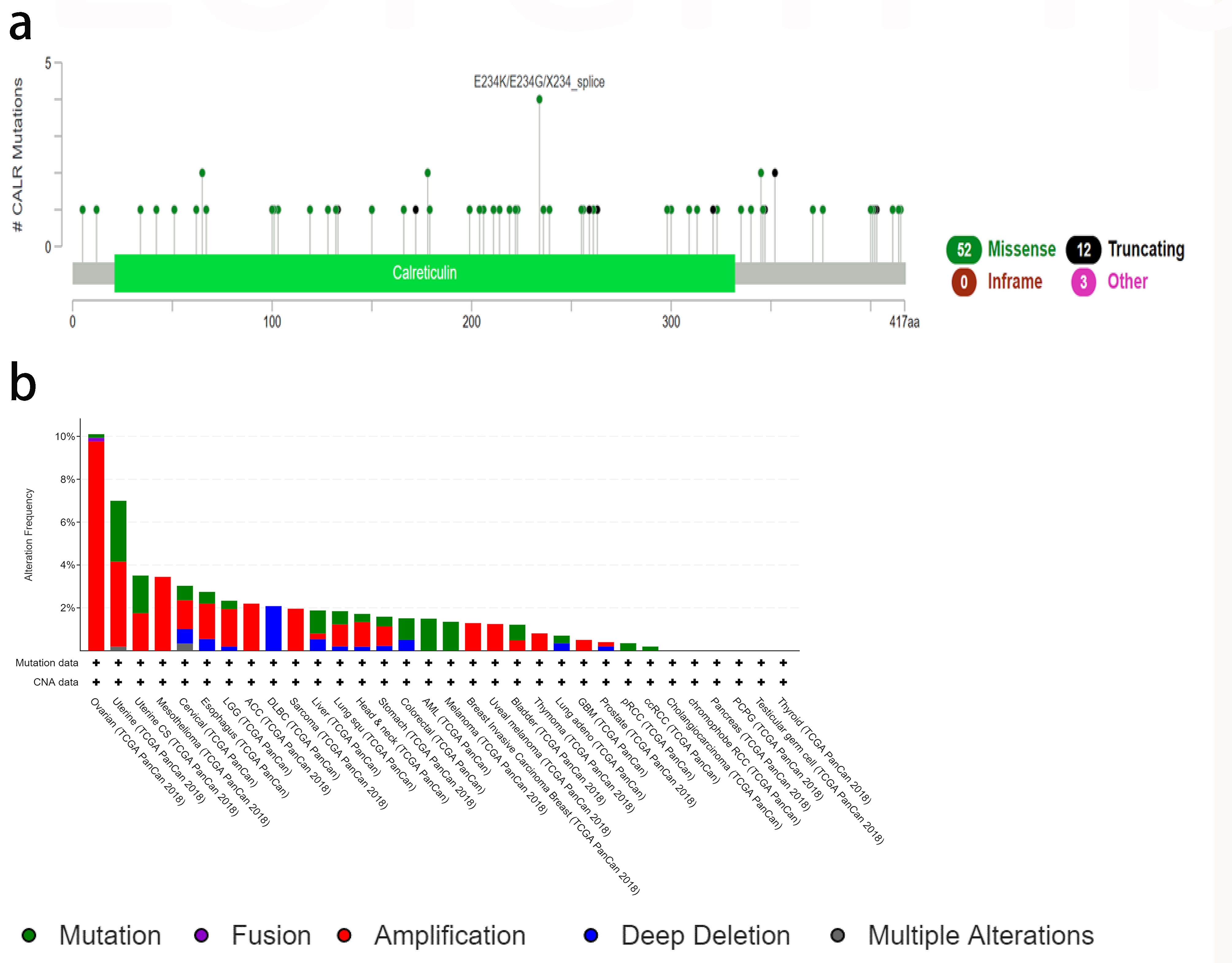

Supplement: S2 Fig — (a) Mutation diagram of CALR across protein domains in different cancer types. (b) Mutation level of CALR in the TCGA database. (TIF) [file pone.0261254.s002.tif]

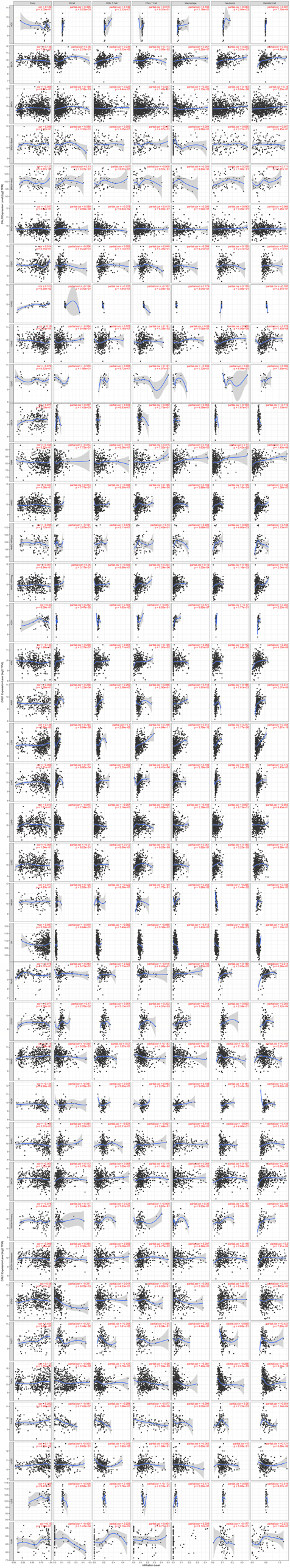

Supplement: S3 Fig — (TIF) [file pone.0261254.s003.tif]
