# Supplementary material for: Bioinformatics analysis for the role of CALR in human cancers
Source: PLoS One. 2021 Dec 15;16(12):e0261254. doi: 10.1371/journal.pone.0261254 (PMC8673678; doi:10.1371/journal.pone.0261254)
Supplement: Attachment — Submitted filename: comment PONE-D-21-19634.docx [file pone.0261254.s006.docx]

Comment

The manuscript titled “Bioinformatics analysis for the role of CALR in human cancers” by Yijun Li et al, aimed to discuss the comprehensive role of CALR in human cancers. Through database mining and bioinformatics analysis, the authors investigated the relationship of CALR expression to a variety of cancers, including pathological stages, RFS, CALR mutations, Genome-wide association analysis, Co-expression analyses and immune cell infiltration, and they concluded that in different tumors CALR expression led to different prognosis.

This study utilizes multiple bioinformatics methods, however it lacks logical connections between analyses. Also the conclusion needs to be experimental validated. Comments are the following:

1. Line 146 Page 7, “the tissues with upregulated CALR mRNA in all three databases”. The “upregulated” is confusing, cause it is not clear what the author are comparing and what is set as control here.
2. Line 155 Page 8, The list of tumors seems redundant. It is more appropriate to show it in the table. In addition, the abbreviation for the tumor name is repeated in the figure legend (Figure 2 & 3).
3. Line 213 Page 10, it makes no sense to enumerate the percentage of each mutation in the different tumors and is lengthy. What is the conclusion for this part? And please use table for data presentation.
4. Line 213 Page 10, “CALR can be detected to be related to other genes”. The statement is ambiguous. What is the relationship and What are the other genes?
5. Line 252 Page 12, PPI and co-expressed proteins are two independent aspects of protein studies. It is unreasonable to summarize them together.
6. In Figure 8, what are the criteria for determining whether CALR expression is related to immune cell infiltration? Moreover, does the CALR gene expression here refer to the tumor cells or the immune cells?
7. The writing of this manuscript needs to be improved. Errors in grammar and typing need to be corrected. Besides, a colloquial expression should be avoided and statements is more accurate in written scientific language.
8. The resolution of the figures needs to be improved.
9. Overall, the data in this study was numerous, but did not reach a clear conclusion in the end. I would suggest that CALR gene should be studied in one specific tumor and validated experimentally.
